# Phytotherapy in Sports Performance and Recovery: A Bibliometric Mapping of Research Themes and Trends

**DOI:** 10.3390/sports14060255

**Published:** 2026-06-22

**Authors:** Amr Chaabeni, Wissem Dhahbi, Ahlem Aissa, Medina Srem-Sai, John Elvis Hagan, Amine Kalai, Vlad Adrian Geantă, Sana Salah, Bassem Charfeddine, Karim Chamari, Anis Jellad

**Affiliations:** 1 Department of Physical Medicine and Rehabilitation, Faculty of Medicine, University of Monastir, Fattouma Bourguiba University Hospital, Monastir 5000, Tunisia; amrch97@gmail.com (A.C.); amine.kalai.1@gmail.com (A.K.); sanasalahdoc@gmail.com (S.S.); 2 Research Laboratory of Technology and Medical Imaging—LR12ES06, Center for Musculoskeletal Biomechanics Research, Faculty of Medicine, University of Monastir, Monastir 5000, Tunisia; anisjellad@gmail.com; 3 Research Unit “Sport Sciences, Health and Movement”, High Institute of Sports and Physical Education of Kef, University of Jendouba, Kef 7100, Tunisia; wissem.dhahbi@gmail.com; 4 Training Department, Police College, Qatar Police Academy, Doha P.O. Box 18624, Qatar; 5 Regional Hospital of Enfidha, Sousse 4030, Tunisia; dr.aissa@gmail.com; 6 Department of Health, Physical Education, Recreation and Sports, University of Education, Winneba P.O. Box 25, Ghana; mssai@uew.edu.gh; 7 Department of Health, Physical Education and Recreation, University of Cape Coast, Cape Coast PMB TF0494, Ghana; 8 Neurocognition and Action-Biomechanics Research Group, Faculty of Psychology and Sports Science, Bielefeld University, 33615 Bielefeld, Germany; 9 Department of Physical Education and Sport, Faculty of Physical Education and Sport, Aurel Vlaicu University of Arad, 310130 Arad, Romania; 10 Department of Biochemistry, Faculty of Medicine, University of Sousse, Sousse 4002, Tunisia; charfeddinebassem@yahoo.fr; 11 Research Department, Naufar, Wellness and Recovery Centre, Doha P.O. Box 93097, Qatar; karim.chamari@naufar.com; 12 Department of Physical Medicine and Rehabilitation, Haj Ali Soua Hospital of Ksar Helal, Faculty of Medicine, University of Monastir, Monastir 5000, Tunisia

**Keywords:** athletic injuries, athletic performance, botanicals, dietary supplements, herbal medicine, phytochemicals, plant extracts, polyphenols

## Abstract

This bibliometric study examines the intellectual structure, evolution, and collaboration patterns of phytotherapy research within sports science to identify key themes and research gaps. Publications indexed in the Web of Science Core Collection from 1991 to 2024 were analyzed using a search strategy combining phytotherapy and sports medicine terms, yielding 3404 records, of which 368 met the inclusion criteria after systematic screening. Performance analysis assessed publication trends, citation impact, and author productivity, while science mapping techniques—including keyword co-occurrence, bibliographic coupling, and co-authorship network analysis—were conducted using Bibliometrix and VOSviewer. Thematic positioning was evaluated through Callon’s centrality-density framework. Results indicate steady growth in the field, with a CAGR of 11.83% and peak output in 2021, involving 2103 authors across 199 sources. International collaboration reached 22.55%, led by the United States, United Kingdom, and China. Dominant research themes include exercise, inflammation, oxidative stress, and phytochemicals such as curcumin and resveratrol. Thematic mapping highlights exercise performance and supplementation as central topics. Overall, the field demonstrates significant expansion, though increased international collaboration and clinical translation are needed.

## 1. Introduction

Sports-related injuries represent a substantial public health and economic burden, affecting athletes across competitive levels and imposing significant constraints on performance capacity, career longevity, and quality of life [1,2,3]. The therapeutic management of musculoskeletal trauma associated with athletic activity has traditionally centered on pharmacological interventions, physical rehabilitation protocols, and surgical interventions when indicated [4,5]. However, concerns regarding the adverse effects of chronic non-steroidal anti-inflammatory drug use, the escalating financial costs of conventional treatment modalities, and growing athlete interest in complementary therapeutic approaches have prompted investigation of alternative interventions [6,7,8]. One of these approaches, phytotherapy, the therapeutic application of plant-derived bioactive compounds, has emerged as a subject of increasing scientific scrutiny within sports medicine contexts [9].

The integration of botanical medicine into athletic health management reflects broader shifts in complementary and alternative medicine utilization across healthcare systems [9]. Plant-derived supplements and herbal preparations have gained traction among athletic populations seeking to optimize recovery kinetics, attenuate exercise-induced inflammation, and mitigate oxidative stress associated with intensive training regimens [10]. Furthermore, athletes must maintain critical awareness regarding supplement composition to avoid inadvertent doping and health risks [11,12]. This concern is particularly relevant for cannabidiol (CBD)-containing products, which are increasingly used to support recovery and anxiety management in athletes. Despite the removal of CBD from the prohibited list, contamination with tetrahydrocannabinol (THC) remains a significant challenge, potentially leading to positive doping tests and unintended ergolytic effects. Longitudinal anti-doping surveillance data confirm increasing THC-positive findings in elite athletes following regulatory changes [11], underscoring the need for rigorous quality control, transparent labeling, and evidence-based guidelines to ensure the safe and responsible use of phytotherapeutic interventions in sport [12]. Epidemiological data indicate substantial prevalence of herbal supplement consumption among gymnasium attendees and competitive athletes [13], with formulations containing botanicals such as curcuma longa, resveratrol-rich extracts, and cannabidiol preparations achieving widespread commercial distribution.

Experimental and clinical investigations have begun to elucidate the bioactive mechanisms through which specific phytochemicals may influence athletic recovery and performance. Curcumin, the principal polyphenolic constituent of turmeric, has demonstrated capacity to modulate exercise-induced muscle damage, inflammatory cascades, and oxidative stress markers in physically active populations, as evidenced by systematic reviews and meta-analyses [14,15]. Randomized controlled trials have documented significant reductions in delayed-onset muscle soreness following curcumin supplementation in athletes subjected to eccentric exercise protocols [16]. Similarly, resveratrol has exhibited potential for enhancing exercise performance and skeletal muscle oxidative capacity through mitochondrial function augmentation [17,18]. Emerging evidence regarding cannabidiol’s anti-inflammatory, analgesic, and neuroprotective properties has stimulated investigation of its applications in sports medicine, particularly concerning muscle regeneration following intensive resistance training [19,20]. Polyphenolic compounds derived from sources including grape extracts, blackcurrant, and pomegranate have demonstrated favorable effects on oxidative stress biomarkers and immune function parameters in exercise contexts [21,22,23]. Additional phytochemicals under investigation include dietary nitrate from beetroot preparations [24,25], ginger extracts for analgesic properties [26], and botanical constituents targeting immune modulation in exercise-induced immunodepression [27,28,29].

Despite accumulating experimental evidence, the phytotherapy literature in sports medicine remains dispersed across multiple disciplinary domains, publication outlets, and methodological frameworks. The absence of comprehensive synthesis regarding research trends, thematic evolution, collaboration networks, and knowledge architecture within this domain limits strategic research planning and evidence-based practice implementation. Bibliometric analysis provides a systematic methodological approach for addressing these limitations. This quantitative technique enables objective assessment of scientific production patterns, citation impact metrics, collaborative relationships among researchers and institutions, and intellectual structure mapping through network-based visualization approaches [30]. Bibliometric methods have been increasingly applied to characterize research domains, identify influential publications and prolific contributors, detect emerging themes, and reveal knowledge gaps requiring investigation [31,32,33]. The application of performance analysis and science mapping techniques to phytotherapy research in sports contexts can illuminate the field’s developmental trajectory, geographic distribution of scientific activity, and thematic clusters representing distinct research foci [34].

The aim of this study was to establish a comprehensive bibliometric profile of phytotherapy research in sports contexts, identify knowledge foundations and emerging trends, characterize collaborative dynamics, and inform future research directions through evidence-based assessment of thematic maturity and field integration.

## 2. Materials and Methods

### 2.1. Study Design

This investigation adopted a bibliometric approach, integrating quantitative indicators of scientific production with qualitative mapping of thematic structures. The methodology was guided by established practices in bibliometric and science mapping research [30,31,33].

### 2.2. Data Source and Search Strategy

#### 2.2.1. Database Selection

The Web of Science (WoS) Core Collection was chosen as the data source because it offers consistent metadata, rigorous journal indexing, and reliable citation tracking. These features make it particularly suitable for advanced bibliometric analyses [35]. In addition, WoS includes high-impact outlets relevant to sports sciences, complementary medicine, and pharmacology [36]. Moreover, as highlighted by Donthu et al. [30], using a single comprehensive database avoids the methodological challenges of merging heterogeneous datasets from multiple sources, reduces the risk of human error, and ensures cleaner, more reliable data for bibliometric evaluation. While WoS provides rigorous indexing, restricting data to a single database may exclude relevant clinical studies indexed exclusively in PubMed or Scopus.

#### 2.2.2. Search Query

A Boolean query was developed to capture publications at the interface of phytotherapy and sport. The final search string was: TS = ((“phytotherapy” OR “herbal medicine” OR “botanical medicine” OR “plant extract*” OR “plant-based therap*” OR “medicinal plant*” OR “natural product*” OR “herbal supplement*” OR “botanical supplement*” OR “essential oil*” OR “curcumin” OR “ginseng” OR “arnica” OR “turmeric” OR “ginger” OR “cannabidiol” OR “CBD” OR “resveratrol” OR “green tea extract” OR “echinacea”) AND (“sport*” OR “athlete*” OR “physical activit*” OR “sports medicine” OR “sports rehabilitation” OR “performance” OR “injury prevention”)).

The search targeted the Topic field (TS), ensuring that terms appeared in the title, abstract, or author keywords. Truncation (*) was applied to include word variations.

#### 2.2.3. Eligibility Criteria

Documents were considered eligible if they met the following conditions: (i) indexed in the Web of Science Core Collection; (ii) published up to 31 December 2024, as this restriction ensured the analysis of complete calendar years, preventing statistical bias from partial data spanning January to July 2025; (iii) written in English; (iv) classified as original articles or reviews; and (v) explicitly addressing phytotherapy, herbal remedies, or plant-based products in relation to sport, exercise, or athletic performance.

Publications were excluded if they mentioned phytotherapy only peripherally without a sports context, focusing solely on general health with no athletic application. We explicitly excluded editorials, conference abstracts, book chapters, letters, data papers, and retracted articles.

#### 2.2.4. Data Collection and Screening

**Figure 1:**
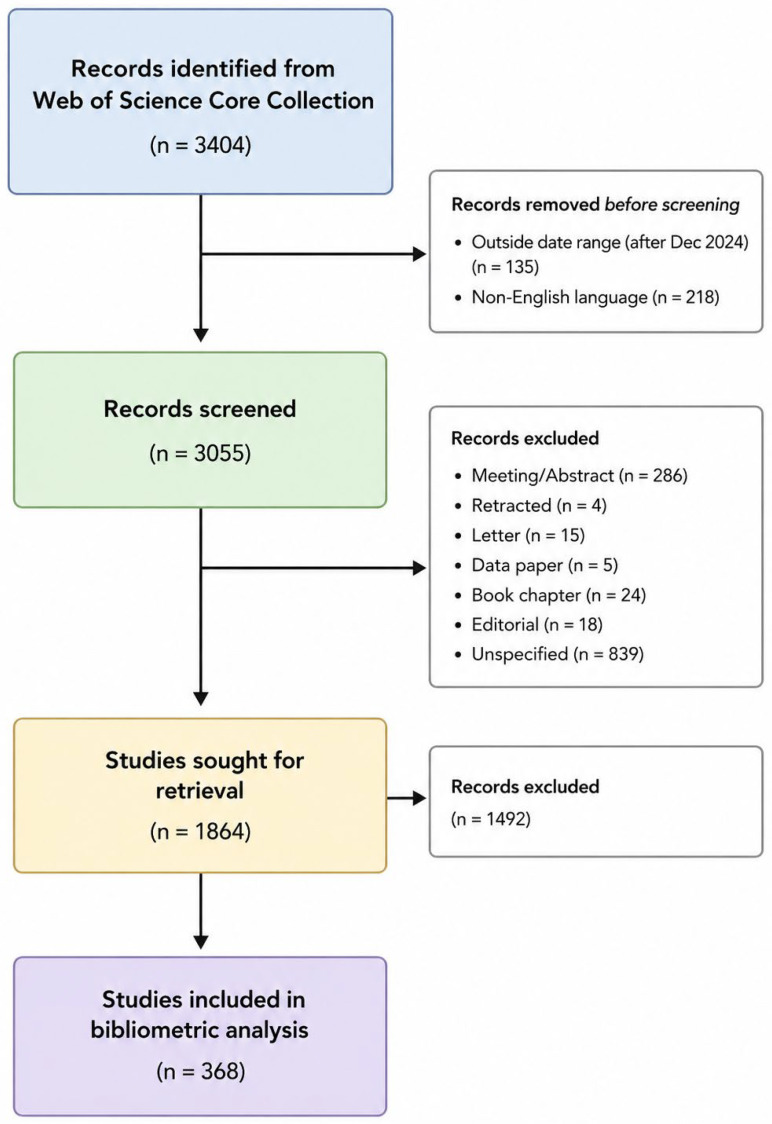
Study flow diagram for systematic literature selection and inclusion criteria application (n = 368 documents from 3404 initial records).

The search was executed on 21 July 2025, identifying 3404 records. After removing non-English records and duplicates, titles and abstracts were screened for relevance. Two independent researchers evaluated relevance during the title and abstract screening phase, resolving any disagreements through consensus. Following this process, 368 publications met the inclusion criteria and were retained for analysis (Figure 1). The unspecified category represents records lacking standard document type metadata in the database, often comprising early access versions, trade journal items, or unclassified correspondence.

Metadata extracted for each document included: title, abstract, author names, keywords (author-provided and KeyWords Plus), year of publication, journal, document type, number of citations, and references cited. Data were exported in plain text and tab-delimited formats with full records and cited references and processed using the Bibliometrix R package (version 4.5.0) [31].

### 2.3. Bibliometric Analysis

To examine the research domain’s intellectual structure, temporal dynamics, and collaborative patterns, we employed complementary bibliometric techniques. The framework integrated performance analysis with thematic mapping in Bibliometrix and network-based science mapping in VOSviewer (version 1.6.20) [37], ensuring methodological rigor and cross-validation.

#### 2.3.1. Performance Indicators

Descriptive indicators were calculated to evaluate publication trends and compound annual growth rate, citation impact (total citations, average citations per document), core journals contributing to the field, author productivity and influence, and institutional and geographical contributions.

#### 2.3.2. Science Mapping

The structural and collaborative dynamics of the field were examined using advanced network analysis techniques. Keyword co-occurrence network was constructed with a threshold of 10 occurrences and normalized by association strength [38]. Bibliographic coupling analysis was conducted applying a minimum link strength of 10 to cluster related publications, and articles with more than 10 citations were included [39]. Also, co-authorship network included authors with at least three publications.

#### 2.3.3. Thematic Mapping

Thematic development and evolution within the field were assessed using Callon’s centrality–density model [40], which positions research themes on a two-dimensional strategic diagram. This approach combines centrality (the degree of interaction a cluster has with other clusters, reflecting its influence on the broader research field) with density (the internal cohesion of a cluster, indicating its stage of development and maturity).

Factorial analysis of author keywords (both Author Keywords and KeyWords Plus) was applied to generate clusters. The analysis was conducted using the Bibliometrix R package, which automatically maps themes into four quadrants of the strategic diagram: (i) Motor themes (high centrality, high density): Well-developed and strongly connected topics that form the intellectual and practical core of the field. These represent research areas that are both mature and influential. (ii) Basic themes (high centrality, low density): Broad, transversal topics that are widely studied but still heterogeneous in their internal development. They often serve as conceptual or methodological foundations for the field. (iii) Niche themes (low centrality, high density): Specialized topics with strong internal cohesion but weak external connections. These represent well-developed but relatively isolated areas of research. (iv) Emerging or declining themes (low centrality, low density): Weakly developed clusters that may represent novel lines of inquiry gaining early attention, or areas losing relevance and gradually disappearing.

This diachronic analysis allowed identification of (i) how phytotherapy-related topics in sport have shifted over time, (ii) which areas have matured into established themes, and (iii) which are emerging as potential new directions for future research.

## 3. Results

### 3.1. General Characteristics of the Included Studies

**Figure 2:**
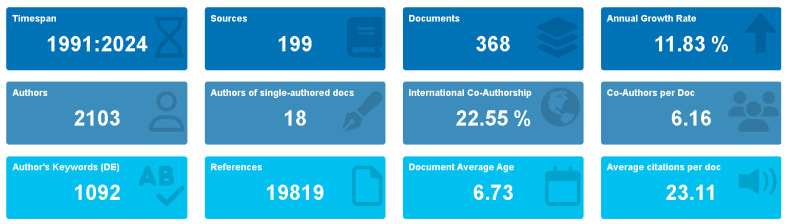
Descriptive bibliometric indicators of the corpus: publication span, authorship metrics, and citation performance (1991–2024).

The included 368 studies, related to phytotherapy use in sports, were published between 1991 and 2024 across 199 sources, with a compound annual growth rate (CAGR) of 11.83%, calculated based on the raw annual publication counts. Our analysis showed the contribution of a total of 2103 authors, with an average number of co-authors per document 6.16 and an international co-authorship frequency of 22.55%. The average age of documents was 6.73 years and the mean citations per document was 23.11 (Figure 2).

### 3.2. Performance Analysis

#### 3.2.1. Scientific Production per Year

**Figure 3:**
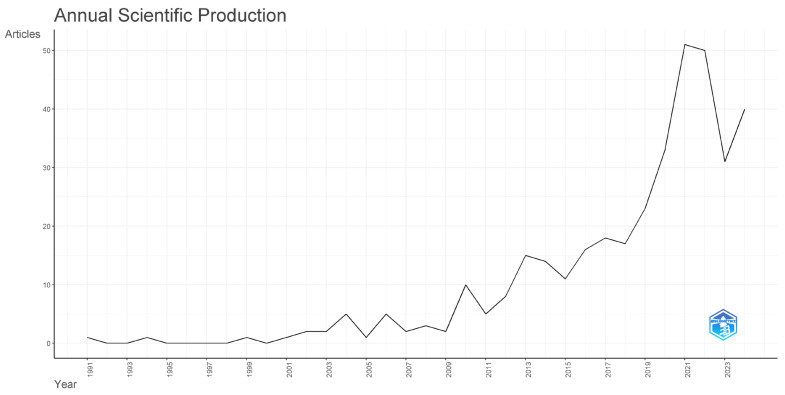
Temporal distribution of scientific production: annual publication frequency and growth trajectory (1991–2024).

The annual scientific production exhibited a gradual increase over the study period, with minimal activity observed between 1991 and the early 2000s. From 2005 onwards, the number of publications showed a steady upward trend, with notable accelerations after 2015. Peak production was reached in 2021, with over 50 articles published (Figure 3).

#### 3.2.2. Most Cited Documents

Citation analysis identified the most influential publications within the corpus based on total citation frequency (Table 1). The top-ranked documents predominantly addressed antioxidant and anti-inflammatory mechanisms of plant-derived bioactive compounds in exercise contexts, with particular emphasis on curcumin, polyphenols, and resveratrol supplementation. Systematic reviews and meta-analyses examining oxidative stress modulation, muscle damage attenuation, and performance enhancement constituted most highly cited works. These foundational publications established methodological frameworks for evaluating phytotherapeutic efficacy in athletic populations and defined key outcome measures subsequently adopted across the field. Citation patterns reflected sustained scholarly engagement with mechanistic investigations of plant extract bioavailability and dose–response relationships in sports medicine applications.

#### 3.2.3. Most Relevant Keywords

**Figure 4:**
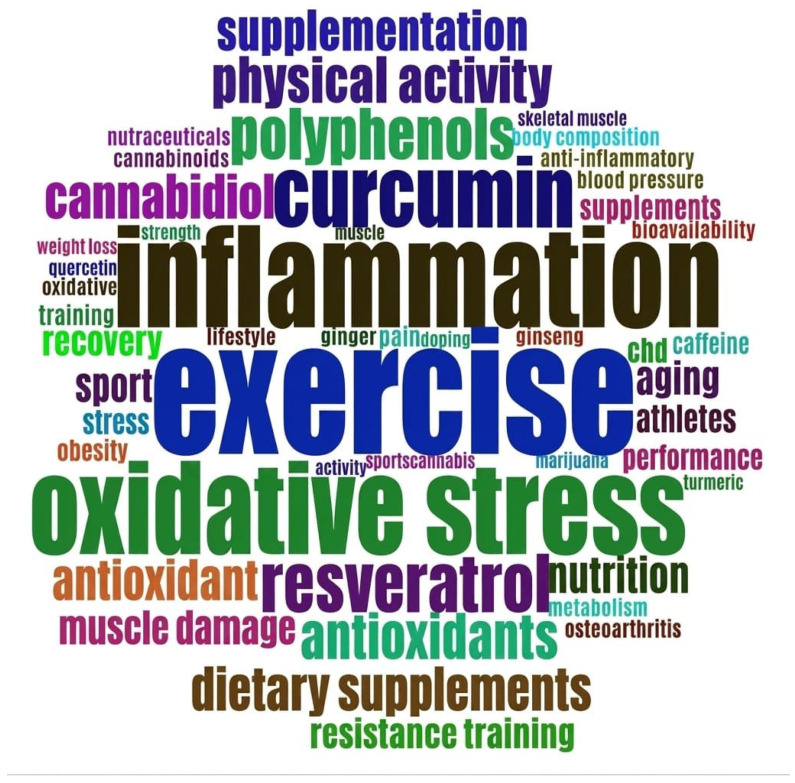
Keyword frequency distribution: most prevalent terms in phytotherapy and sports science intersection (threshold ≥ 10 occurrences).

Keywords that emerged most frequently were “exercise”, “inflammation”, “oxidative stress”, “curcumin”, and “resveratrol” (Figure 4). The most frequent used phytotherapy-related keywords were “curcumin”, “resveratrol”, “polyphenols”, “antioxidant”, and “cannabidiol” (Table 2).

**Figure 5:**
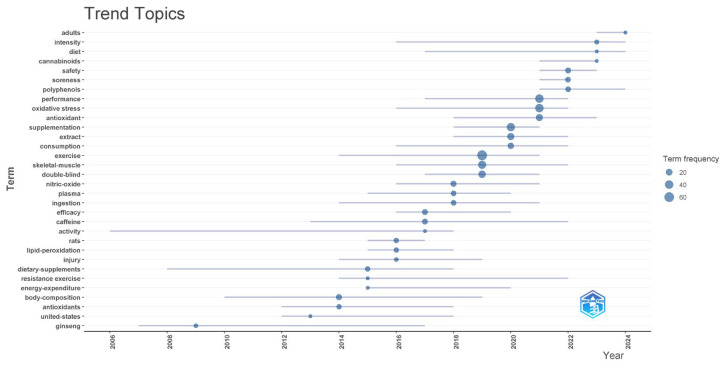
Longitudinal keyword evolution: temporal emergence and persistence of phytotherapy-related research terms (2000–2024).

Figure 5 presents the temporal evolution of keywords related to phytotherapy use in sport, as identified in the bibliometric analysis. Each horizontal line represents the timespan of a given keyword, while the size of the circles reflects its frequency of occurrence. Earlier terms such as ginseng, activity, and dietary supplements appear before 2010, whereas others, including body composition, caffeine, and antioxidants, gain visibility between 2012 and 2016. From 2017 onward, a wider range of keywords emerges, with exercise, consumption, extract, supplementation, antioxidant, oxidative stress, performance, and polyphenols showing higher frequency, as indicated by larger nodes. More recent entries, including cannabinoids, diet, intensity, and adults, appear around 2021–2023.

#### 3.2.4. Most Relevant Journals

**Figure 6:**
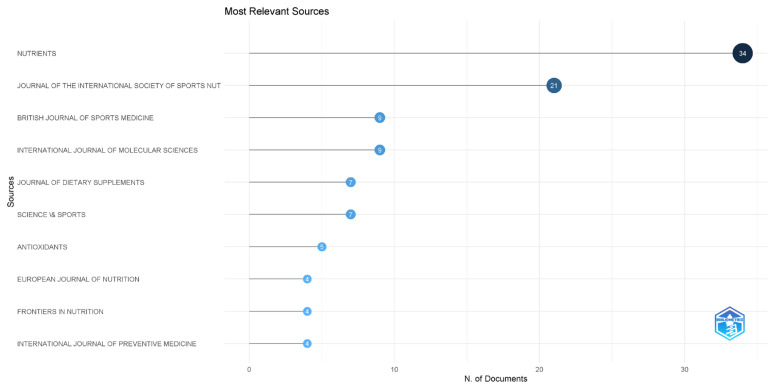
Source journal productivity ranking: core outlets contributing to phytotherapy in sports research (top 10 sources by document count).

The two leading journals were “Nutrients” (34 articles) and “Journal of the “International Society of Sports Nutrition” (21 articles) (Figure 6). 

#### 3.2.5. Most Relevant Authors

The two most relevant authors, according to the number of publications, were Burke, Louise M and Stear, Samantha with 7 articles each (Table 3).

#### 3.2.6. Most Relevant Authors’ Affiliations and Countries

**Figure 7:**
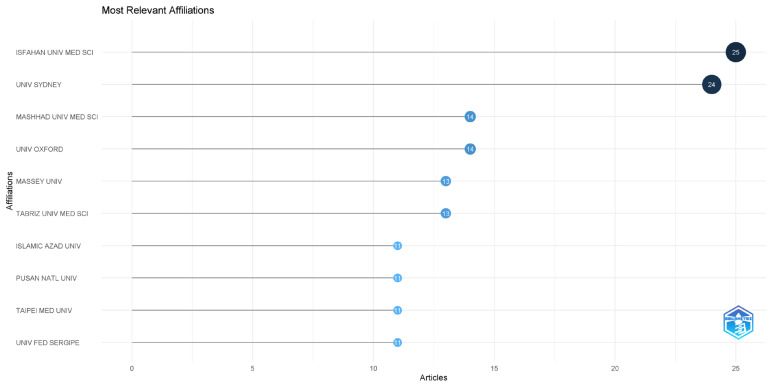
Institutional productivity distribution: leading academic affiliations by document contribution (top 10 institutions).

The most relevant authors’ affiliations were ‘Isfahan University of Medical Sciences’ with 26 articles and ‘University of Sydney’ with 24 articles (Figure 7).

**Figure 8:**
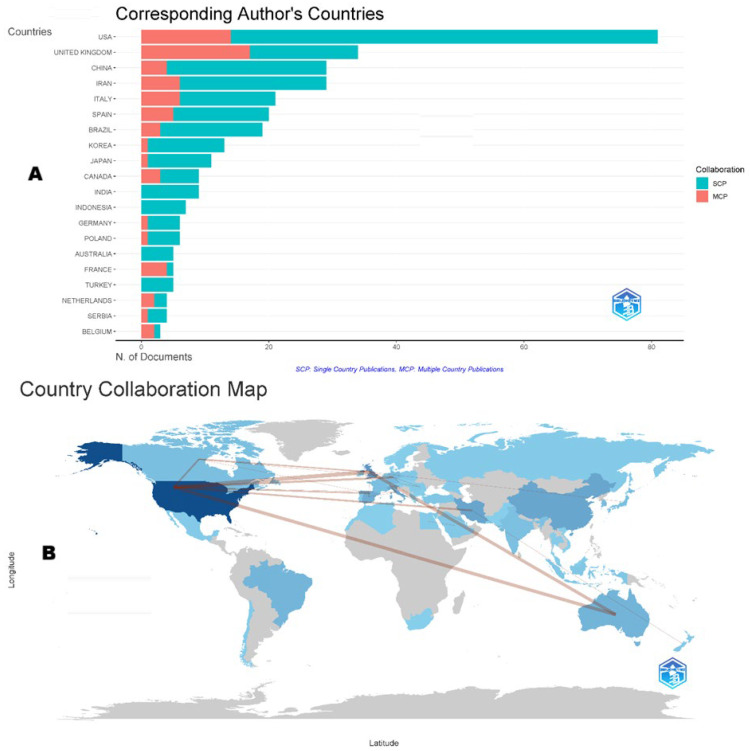
Geographic distribution and international collaboration networks: (**A**) corresponding author country productivity; (**B**) inter-country collaboration intensity map.

The bar chart (Figure 8A) highlights the dominance of the USA, the UK, and China in corresponding authorship, with the USA leading substantially. The collaboration map (Figure 8B) complements this by visualizing international linkages. It shows the USA as the primary hub of global research collaboration, extending ties to Europe, Asia, and Australia.

### 3.3. Scientific Mapping

#### 3.3.1. Keywords Network

**Figure 9:**
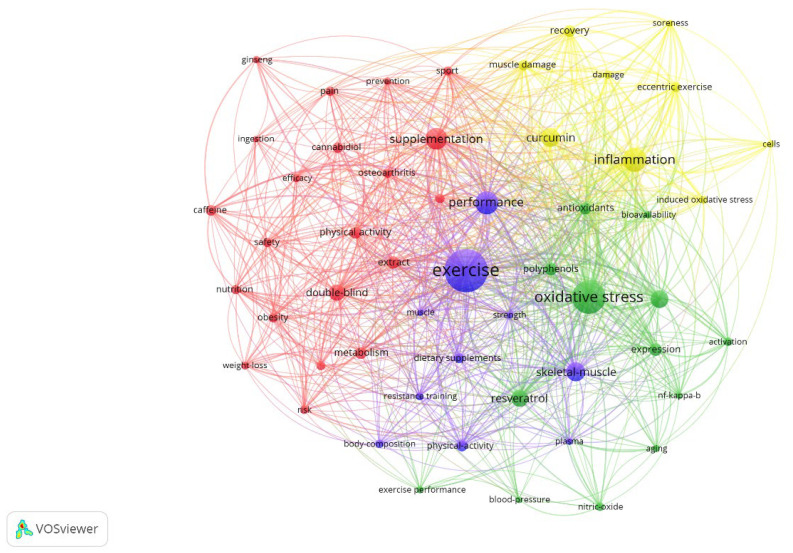
Keyword co-occurrence network analysis: thematic clustering and semantic relationships (minimum occurrence threshold = 10; association strength normalization).

Figure 9 shows a keyword co-occurrence network where node size reflects the frequency of use of each keyword, and colors represent distinct clusters. The red cluster contains terms such as supplementation, performance, double-blind, nutrition, cannabidiol, caffeine, obesity, safety, ingestion, extract, weight loss, physical activity, pain, and osteoporosis. The yellow cluster includes inflammation, curcumin, muscle damage, recovery, cells, eccentric exercise, soreness, damage, and bioavailability. The green cluster is composed of oxidative stress, skeletal muscle, resveratrol, antioxidants, plasma, polyphenols, expression, activation, NF-κB, and nitric oxide. The blue cluster contains exercise, performance, strength, muscle, resistance training, dietary supplements, and extract. The purple cluster includes body composition, exercise performance, physical activity, metabolism, nutrition, and weight loss.

#### 3.3.2. Authors Collaboration Network

**Figure 10:**
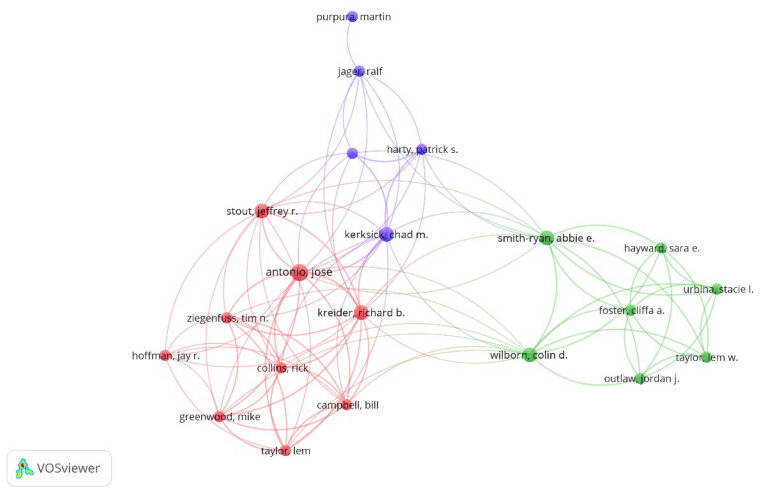
Co-authorship collaboration network: author clustering and collaborative linkages (minimum publication threshold = 3 documents per author).

The authorship collaboration network analysis revealed three distinct clusters. Cluster 1 (red) included Antonio J., Stout J.R., and Kreider R.B. as the main contributors. In Cluster 2 (blue), Kerksick C.M. emerged as the primary contributor. Cluster 3 (green) was represented mainly by Wilborn C.D. and Smith-Ryan A.E. as the leading contributors (Figure 10).

#### 3.3.3. Bibliographic Coupling Network

**Figure 11:**
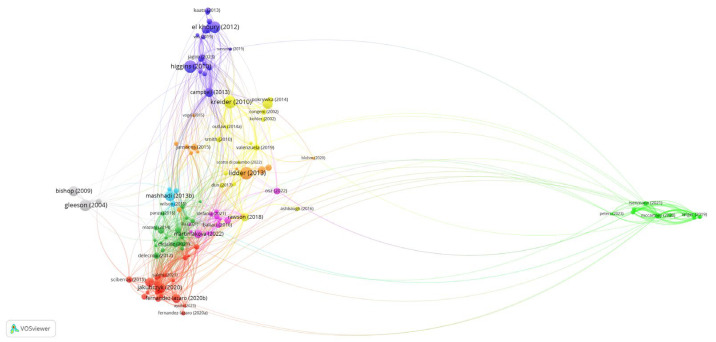
Bibliographic coupling network: document clustering based on shared reference patterns (minimum link strength = 10; minimum citation threshold = 10).

The bibliographic coupling analysis revealed nine distinct clusters (Figure 11). Cluster 1 (red) includes publications by Jakubczyk et al. [15], Sciberras et al. [46], Fernández-Lázaro et al. [14,47], Sorrenti et al. [48], and Drobnic et al. [16]. Cluster 2 (green) consists of Elejalde et al. [21], Cook et al. [49], Ruiz-Iglesias et al. [22], and Giménez-Bastida et al. [23]. Cluster 3 (blue) comprises El Khoury et al. [13], Higgins et al. [44], Campbell et al. [50], and Kaats et al. [51]. Cluster 4 (yellow) contains Kreider et al. [42], Bahrke et al. [45], Rawson et al. [10], Outlaw et al. [52], and Valenzuela et al. [53]. Cluster 5 (pink) includes Martiniakova et al. [54], Sung et al. [17], and Baltaci et al. [18]. Cluster 6 (light green) is formed by McCartney et al. [19], Isenmann et al. [20], Zeiger et al. [55], and Jîtcă et al. [56]. Cluster 7 (orange) encompasses Lidder et al. [24], Rimer et al. [57], and Wylie et al. [25]. Cluster 8 (light blue) includes Wilson et al. [26] and Mashhadi et al. [44]. Cluster 9 (grey) consists of Gleeson et al. [27,28] and Bishop et al. [29].

#### 3.3.4. Strategic Thematic Map (Callon’s Centrality–Density Framework)

The thematic map analysis using Callon’s centrality–density framework identified four clusters of research themes.

**Figure 12:**
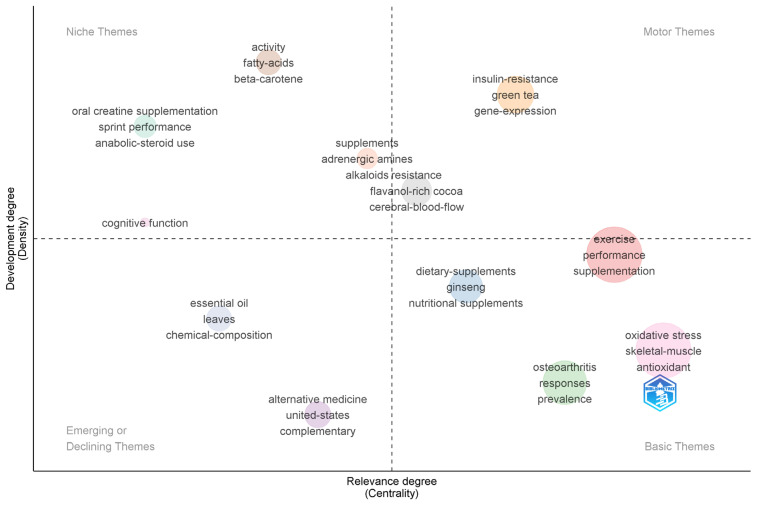
Strategic thematic map based on Callon’s centrality–density framework: positioning of research themes by development stage and field integration.

In the motor themes quadrant (high centrality and high density), key topics included “exercise performance” and “supplementation”, as well as “insulin resistance”, “green tea”, and “gene expression”. In the basic themes quadrant (high centrality but low density), dominant themes were “oxidative stress”, “skeletal muscle”, “antioxidant”, “osteoarthritis”, “responses”, and “prevalence”. The niche themes quadrant (high density but low centrality) encompassed “oral creatine supplementation”, “sprint performance”, “anabolic-steroid use”, “activity”, and “beta-carotene”. Finally, the emerging or declining themes quadrant (low centrality and low density) included “alternative medicine”, “complementary medicine”, “essential oil”, “chemical composition”, and “leaves” (Figure 12).

## 4. Discussion

This bibliometric investigation identified 368 publications spanning three decades (1991–2024) that constitute the research corpus at the intersection of phytotherapy and sports science. The CAGR of 11.83% reflects sustained scholarly interest in plant-derived therapeutic interventions for athletic populations, with production accelerating substantially after 2015 and reaching peak output in 2021 with over 50 publications. This temporal pattern aligns with broader trends in complementary and alternative medicine research, where evidence-based evaluation of botanical supplements has intensified in response to market proliferation and athlete demand [10]. The 2103 contributing authors and average collaboration index of 6.16 co-authors per document indicate robust engagement across research teams, though the international co-authorship rate of 22.55% suggests that cross-border collaboration remains moderate relative to other biomedical research domains where international partnerships frequently exceed 40% [30]. The concentration of output within two leading journals (Nutrients with 34 articles and Journal of the International Society of Sports Nutrition with 21 articles) establishes these outlets as primary dissemination channels for phytotherapy-related sports research, likely reflecting their interdisciplinary scope bridging nutrition science, exercise physiology, and clinical investigation.

The geographic distribution of research activity reveals clear regional specialization, with the United States, United Kingdom, and China constituting primary production hubs. The collaborative network analysis demonstrated that the United States functions as the central node in international partnerships, extending linkages across Europe, Asia, and Australia. This pattern reflects established research infrastructure concentrations, funding availability for dietary supplement investigations, and historical strengths in sports science research within these regions. The identification of Isfahan University of Medical Sciences and University of Sydney as leading institutional contributors (26 and 24 publications respectively) highlights specific centers of excellence where sustained programmatic research on phytotherapeutic interventions has developed. These findings provide strategic intelligence for identifying potential collaboration partners and understanding regional research priorities within the field.

Keyword co-occurrence analysis revealed that the intellectual structure of phytotherapy research in sports contexts organizes around five primary thematic clusters. The prominence of terms including exercise, inflammation, oxidative stress, curcumin, and resveratrol in frequency distributions indicates that antioxidant and anti-inflammatory mechanisms constitute the dominant conceptual framework guiding investigation in this domain. This mechanistic focus is scientifically justified given that strenuous physical activity induces oxidative stress through reactive oxygen species generation, mitochondrial electron transport chain disruption, and inflammatory cytokine release [27]. The temporal keyword evolution analysis demonstrated that earlier research (pre-2010) concentrated on traditional botanicals including ginseng and broad categories such as dietary supplements, whereas contemporary investigations increasingly target specific bioactive compounds with defined molecular mechanisms. The recent emergence of cannabidiol, polyphenols, and beetroot-derived nitrates as research foci reflects both evolving regulatory landscapes and advances in phytochemical characterization methodologies that enable precise dose–response evaluations.

The bibliographic coupling analysis identified nine distinct research clusters, each representing coherent thematic areas with shared reference patterns. Cluster 1, encompassing studies by Jakubczyk et al. [15], Fernández-Lázaro et al. [14,47], Drobnic et al. [16], and Sorrenti et al. [48], centers on curcumin supplementation and its effects on exercise-induced muscle damage, inflammation, and recovery kinetics. The concentration of systematic reviews and meta-analyses within this cluster suggests that curcumin research has achieved sufficient empirical density to support evidence synthesis, with documented effects on delayed-onset muscle soreness reduction and inflammatory marker modulation [47]. Cluster 2, containing investigations by Elejalde et al. [21], Ruiz-Iglesias et al. [22], and Giménez-Bastida et al. [23], examines polyphenolic compounds from diverse botanical sources including grape, blackcurrant, and pomegranate extracts. The mechanistic rationale supporting this research direction derives from polyphenols’ capacity to attenuate oxidative stress through direct free radical scavenging, upregulation of endogenous antioxidant enzyme systems, and modulation of redox-sensitive transcription factors including nuclear factor kappa B [48].

Cluster 4, comprising studies by Kreider et al. [42], Valenzuela et al. [53], and related investigations, represents a broader examination of multi-ingredient supplementation strategies and ergogenic aids that extend beyond strictly botanical interventions. This cluster’s position within the bibliographic coupling network suggests conceptual adjacency between phytotherapy research and the wider sports nutrition literature addressing performance enhancement and recovery optimization. Cluster 6, containing recent publications by McCartney et al. [19], Isenmann et al. [20], and Jîtcă et al. [56], reflects the emerging cannabidiol research trajectory. The inclusion of systematic reviews within this cluster indicates rapid evidence accumulation regarding cannabidiol’s potential applications in sports medicine, particularly concerning anti-inflammatory, analgesic, and muscle regeneration properties [19,56]. Cluster 7, comprising investigations of dietary nitrate from beetroot preparations [24,25], examines nitric oxide pathway modulation as a mechanism for enhancing endurance performance through improved oxygen delivery and mitochondrial efficiency.

The thematic mapping analysis based on Callon’s centrality-density framework positioned exercise performance and supplementation as motor themes, characterized by high centrality and density. This classification indicates that these topics represent well-developed research areas with strong internal coherence and substantial external connections to other themes within the field. The positioning of oxidative stress and skeletal muscle as basic themes (high centrality, low density) suggests that while these concepts are widely studied and conceptually central to the field, they remain heterogeneous in their development and lack the internal cohesion characteristic of mature research domains. The identification of oral creatine supplementation, sprint performance, and anabolic steroid use as niche themes (high density, low centrality) reflects specialized research areas with strong internal development but limited integration with broader field concerns. The classification of alternative medicine, complementary medicine, and essential oils as emerging or declining themes (low centrality, low density) warrants careful interpretation, as it may indicate either nascent research areas gaining initial attention or diminishing topics losing relevance within contemporary sports science priorities. Compared with previous bibliometric studies focusing on specific compounds such as curcumin or cannabidiol [58,59], the present analysis provides a broader macro-level synthesis of sports-related phytotherapy. It highlights the convergence of key thematic domains, including antioxidant activity, inflammation-related pathways, and dietary supplementation, which have also been identified in bibliometric research on nutritional supplements in sport and exercise [60]. This integrative perspective underscores the conceptual overlap between distinct botanical and nutraceutical interventions within sports and exercise science.

Several methodological strengths characterize this investigation. The exclusive use of WoS Core Collection ensured consistent metadata quality, reliable citation tracking, and access to high-impact publications relevant to sports sciences and complementary medicine [35]. The comprehensive temporal scope (1991–2024) enabled detection of longitudinal trends and evolutionary patterns in the research focus. The integration of multiple bibliometric techniques, including performance analysis, keyword co-occurrence networks, bibliographic coupling, co-authorship analysis, and thematic mapping, provided multidimensional characterization of the research landscape. The application of established normalization methods (association strength for keyword co-occurrence, minimum threshold criteria for network inclusion) enhanced analytical rigor and interpretability of network visualizations [37,39].

### 4.1. Limitations and Future Perspectives

However, several limitations require acknowledgment. The restriction to English-language publications indexed in WoS may have excluded relevant research published in other languages or regional journals, potentially introducing geographic bias. The reliance on author-provided keywords and KeyWords Plus for thematic analysis is susceptible to inconsistencies in keyword assignment practices across authors and time periods. Bibliographic coupling analysis reflects shared reference patterns rather than direct conceptual overlap, and temporal lags between publication of cited works and citing publications may influence cluster compositions. Furthermore, relying exclusively on WoS may omit clinically valuable studies predominantly indexed in PubMed. The current corpus lacks a rigorous quality appraisal of the included literature, meaning the ratio of randomized controlled trials to observational studies remains unquantified. The absence of full-text analysis limits detection of nuanced thematic relationships that may not be captured through keyword co-occurrence or reference patterns alone. A limitation of this study is that author keywords in the co-occurrence network were analyzed in their original form without full synonym unification (e.g., “cannabidiol” and “CBD”), as the software workflow used does not allow thesaurus-based merging without altering the underlying co-occurrence structure; this may have introduced minor fragmentation in the visualization of thematic clusters. Citation-based metrics reflect scholarly impact within academic contexts but do not directly measure clinical translation, practical implementation, or real-world effectiveness of investigated interventions. Additionally, variations in author name indexing may artificially fragment individual productivity metrics despite standard data normalization efforts. Future research should employ qualitative or mixed-methods approaches to investigate specific substance trajectories over time, identifying which phytochemicals represent emerging clinical trends versus historical artifacts.

### 4.2. Practical Implications

Research institutions and funding agencies are encouraged to prioritize international collaboration initiatives to elevate the current 22.55% co-authorship rate, particularly facilitating partnerships between established centers in North America, Europe, and emerging research hubs in Asia. Investigators should target mechanistic investigations that integrate molecular biology, metabolomics, and exercise physiology methodologies to elucidate precise pathways through which phytochemicals modulate exercise-induced oxidative stress, inflammatory responses, and recovery processes. Standardized reporting protocols for phytotherapeutic interventions, including botanical source verification, bioactive constituent quantification, bioavailability assessment, and dose–response characterization, require development and adoption to enhance reproducibility. Translation of experimental findings into clinical practice necessitates rigorous randomized controlled trials with athlete populations, incorporating sport-specific performance outcomes, long-term safety monitoring, and personalized dosing strategies informed by pharmacogenomic considerations. Clinicians must navigate the significant heterogeneity of supplementation protocols and the variable quality of commercial products. Practitioners should prioritize interventions backed by human clinical trials over historical traditional uses. Sports medicine practitioners should maintain evidence-based skepticism regarding commercial phytotherapy products until substantiated by peer-reviewed investigations meeting established quality standards.

## 5. Conclusions

This bibliometric analysis documented sustained growth in phytotherapy research within sports science contexts, with 368 publications demonstrating 11.83% annual expansion and thematic organization around oxidative stress mitigation, inflammation modulation, and performance enhancement. Geographic concentration in the United States, United Kingdom, and China, combined with moderate international collaboration (22.55%), indicates opportunities for expanded cross-border partnerships. The intellectual structure encompasses nine bibliographic coupling clusters addressing curcumin, polyphenols, cannabidiol, dietary nitrates, and immune function, with motor themes centered on exercise performance and supplementation. Future investigations should prioritize mechanistic elucidation, standardized intervention reporting, and rigorous clinical translation to transform accumulating empirical evidence into evidence-based practice recommendations for athletic populations.

## Figures and Tables

**Table 1:** Most cited documents: top-ranked publications by total citation per year count and bibliometric impact indicators.

Paper	Title	Total Citations	Total Citations per Year
Fernández-Lázaro et al. (2020) [14]	Modulation of Exercise-Induced Muscle Damage, Inflammation, and Oxidative Markers by Curcumin Supplementation in a Physically Active Population: A Systematic Review	178	29.67
Jakubczyk et al. (2020) [15]	Antioxidant Potential of Curcumin—A Meta-Analysis of Randomized Clinical Trials	178	29.67
Lidder et al. (2013) [24]	Vascular effects of dietary nitrate (as found in green leafy vegetables and beetroot) via the nitrate-nitrite-nitric oxide pathway	256	19.69
Salamon et al. (2019) [41]	Medical and Dietary Uses of N-Acetylcysteine	132	18.86
Kreider et al. (2010) [42]	ISSN exercise & sport nutrition review: research & recommendations	278	17.38
Higgins et. (2010) [43]	Energy beverages: content and safety	266	16.63
EL Khoury et al. (2012) [13]	Intake of Nutritional Supplements among People Exercising in Gyms in Beirut City	216	15.43
Mashhadi et al. (2013) [44]	Anti-oxidative and anti-inflammatory effects of ginger in health and physical activity: review of current evidence	166	12.77
Gleeson et al. (2004) [27]	Exercise, nutrition and immune function	227	10.32
Bahrke et al. (2004) [45]	Abuse of anabolic androgenic steroids and related substances in sport and exercise	173	7.86

**Table 2:** Phytotherapy-specific keyword frequency analysis: botanical and bioactive compound terminology prevalence in sports science literature.

Terms	Frequency
Antioxidants	28
Curcumin	23
Cannabidiol	22
Resveratrol	19
Polyphenols	17
Dietary supplements	14
Supplementation	13
Nutrition	12
Supplements	8
Caffeine	7

**Table 3:** Author productivity ranking: leading contributors by publication count and proportional output distribution.

Authors	N Articles	H-Index
Burke, Louise M	7	77
Stear, Samantha	7	12
Castell, Lindy	6	21
Smith, Abbie	5	46
Askari, Gholamreza	4	38
Wilborn, Colin	4	29
Del Coso, Juan	4	45
Shokri-Mashhadi, Nafiseh	4	12
Bishop, Nicolette C.	4	40
Antonio, Jose	4	35

## Data Availability

The data that support the findings of this study are derived from the Web of Science Core Collection. The full search strategy is provided in the Methods section. Due to the proprietary nature of the database, raw data cannot be publicly shared; however, the dataset can be reproduced using the described query. Extracted data are available from the corresponding author upon reasonable request.
